# Differential regulation of tissue-resident and blood-derived macrophages in models of autoimmune and traumatic peripheral nerve injury

**DOI:** 10.3389/fimmu.2024.1487788

**Published:** 2024-11-19

**Authors:** Alina Sprenger-Svačina, Martin K. R. Svačina, Tong Gao, Rodney M. Ritzel, Louise D. McCullough, Kazim A. Sheikh, Gang Zhang

**Affiliations:** ^1^ Department of Neurology, McGovern Medical School, The University of Texas Health Science Center at Houston, Houston, TX, United States; ^2^ Department of Neurology, Faculty of Medicine and University Hospital of Cologne, Cologne, Germany

**Keywords:** autoimmune polyneuropathy, traumatic peripheral nerve injury, endoneurial macrophages, blood-derived macrophages, tissue-resident macrophages, macrophage polarization, myelin phagocytosis, cytokine

## Abstract

**Introduction:**

The current study focuses on understanding the functional role of different subsets of endoneurial macrophages in autoimmune polyneuropathies (AP) and traumatic peripheral nerve injury (TPNI), which holds potential for clinical application. Recent studies have advanced our understanding of the diverse origins of macrophages within peripheral nerves. However, there remains a gap in our knowledge regarding how endoneurial macrophages from different origins affect disease progression in AP versus TPNI.

**Methods:**

Flow cytometry was utilized to analyze macrophage phenotypes, including polarization states, cytokine production, and myelin phagocytosis in animal models of AP and TPNI. This study focuses on two distinct origins of macrophages, namely CD11b^+^F4/80^hi^ tissue-resident (TRM) and CD11b^+^F4/80^int^ blood-derived macrophages (BDM). The study utilized two animal models: the first was the spontaneous autoimmune peripheral polyneuropathy (SAPP) model in B7.2-null non-obese diabetic (NOD-B7.2-/-) mice, which serves as a model for inflammatory demyelinating polyneuropathy; the second model involved wild type C57BL/6 mice subjected to sciatic nerve crush injury, modeling TPNI. Behavioral, electrophysiological, and histological analyses were performed to assess peripheral nerve injury.

**Results:**

The study found that pro-inflammatory M1 macrophage polarization and tumor necrosis factor-alpha production by macrophages were more pronounced in the peripheral nerves of SAPP mice compared to those with TPNI, with the majority of these macrophages being TRM. In contrast, endoneurial macrophages in mice with TPNI were mainly BDM, exhibiting a less defined macrophage polarization and cytokine profile than TRM in AP mice. Interestingly, myelin phagocytosis was primarily driven by BDM in both SAPP and TPNI mice.

**Discussion:**

This study offers novel insights into origin-dependent macrophage functions in AP and TPNI. Furthermore, these findings may help the future development of novel therapies targeting macrophage subsets of specific origin in AP and TPNI.

## Introduction

Autoimmune polyneuropathies (AP) and traumatic peripheral nerve injury (TPNI) represent two distinct yet interconnected challenges in the field of peripehral neuropathies. Both conditions entail a complex interplay of cellular and molecular responses, where macrophages emerge as pivotal players in the initiation, progression, and resolution of tissue damage. Among the diverse array of immune cells orchestrating the inflammatory cascade, tissue-resident macrophages (TRM) and blood-derived macrophages (BDM) hold critical roles in shaping the pathophysiological landscape of these disorders (1, 2).

TRM are intrinsic components of the peripheral nervous system (PNS), strategically positioned within nerve tissues to maintain homeostasis and respond promptly to insults. These TRM, deriving either from late embryonic precursors (referred to as long-term TRM) or undergoing intermediate-term replenishment by BDM (2, 3), exert immunomodulatory functions under steady-state conditions, surveilling the microenvironment for signs of disturbance. However, upon encountering inflammatory stimuli or injury, resident macrophages undergo phenotypic shifts and elicit diverse effector responses, ranging from phagocytosis of debris to cytokine secretion and antigen presentation (2, 4–6). Conversely, BDM, originating from circulating monocytes, infiltrate peripheral nerves in response to injury or inflammation, amplifying the local immune response (7, 8). These infiltrating macrophages exhibit remarkable plasticity, adapting their phenotypes and functions based on environmental cues (8, 9). Upon infiltration, BDM contribute to tissue repair and remodeling through phagocytosis of cellular debris, secretion of trophic factors, and modulation of inflammation (9). However, dysregulated or prolonged activation of BDM may exacerbate tissue damage and perpetuate endoneurial inflammation, thereby exacerbating the pathogenesis of peripheral neuropathy and nerve injury.

AP, such as Guillain-Barré syndrome (GBS) and its chronic counterpart, chronic inflammatory demyelinating polyneuropathy (CIDP), can lead to substantial disability via inflammatory demyelination of peripheral nerves (10–13). Besides aberrant B and T cell activation (14–17), macrophages play a pivotal role in nerve damage by presenting autoantigens to T cells, signaling through pro-inflammatory chemokines/cytokines, and engaging in myelin phagocytosis (3, 18–21). While evidence suggests that the endoneurial macrophage response in AP leans towards a pro-inflammatory M1 polarization state (22) and contributes to pathogenic myelin phagocytosis (20, 23), it remains unclear whether: i) endoneurial macrophage populations of diverse origins react differently to the autoinflammatory environment, and ii) the origin of macrophages dictates distinct effector functions in these conditions.

Several studies suggest that in TPNI, acute endoneurial inflammation primarily increases the BDM population within the endoneurium, reaching its peak at 7-10 days post-injury (9). This response is triggered by the disruption of the blood-nerve barrier and the release of macrophage-attracting chemokines such as CX3CL1 and CCL2 from injured axons (2, 3, 9). Following an initial pro-inflammatory M1 macrophage reaction aimed at clearance of endoneurial debris, a shift in macrophage polarization towards an anti-inflammatory and pro-regenerative M2 state becomes crucial for facilitating effective nerve repair (9, 24).

Research into the origins of endoneurial macrophages is complicated by the continual replacement of TRM populations with an influx of BDM, a phenomenon that intensifies following nerve injury. Additionally, the dynamics by which newly recruited endoneurial BDM adapt TRM marker expression remain largely unexplored. As a result, an unequivocal distinction between long-term and intermediate-term TRM through flow cytometric analysis of cellular surface markers has not yet been definitively established. A recent single-cell transcriptomics study found that the majority of BDM reduce or lose expression of Ly6C, a major monocyte/BDM marker, approximately 5-7 days after the initial influx of endoneurial BDM following TPNI (1). Taking this finding into consideration, we opted to discern between BDM and TRM by assessing the level of expression of the common macrophage marker F4/80, which has been used by others for this purpose (25). In our study, we utilize this marker to distinguish between BDM (CD11b^+^F4/80^int^) that likely have infiltrated the nerve within the past seven days, and TRM (CD11b^+^F4/80^hi^), that have been present in the nerve for more than seven days, including the long-term TRM originating from embryonic precursors.

In the current study, we investigate the various roles played by TRM and BDM in murine models of AP and TPNI, highlighting their significance in disease pathogenesis. Given the emerging focus on directly modulating endoneurial macrophages as a targeted therapeutic approach in experimental neuritis models (26, 27), our study, elucidating the distinct macrophage functions associated with their origins in the context of AP and TPNI, may help unveiling novel therapeutic targets aimed at modulating endoneurial inflammation, promoting tissue repair, and restoring neurological function.

## Materials and methods

### Animals

Wild-type C57BL/6 mice were obtained from The Jackson Laboratory (Bar Harbor, ME, USA), and B7.2-null non-obese diabetic mice (NOD-B7.2-/- mice) were bred and raised in our animal facility. The animals were randomly assigned to groups through a computer-generated allocation sequence that remained concealed from researchers until the intervention stage. The study was conducted using a double-blind design, ensuring that both the researchers administering treatments and those assessing outcomes, including behavioral analysis, were unaware of group assignments. All experimental procedures were carried out in compliance with institutional and federal guidelines for animal research. This study was approved by the Animal Welfare Committee at the University of Texas Health Science Center at Houston (AWC-23-0034).

### Spontaneous autoimmune peripheral polyneuropathy animal model

The SAPP mouse model, developed in NOD mice with a deficiency in the costimulatory molecule, B7.2 (NOD-B7.2-/- mice), is pathologically similar to CIDP. It is considered as the most representative animal model for studying AP (28, 29). NOD-B7.2-/- mice spontaneously develop a progressive, symmetric, autoimmune-mediated demyelinating sensorimotor polyneuropathy between 20 and 24 weeks of age, with the condition peaking between 30 and 35 weeks of age (29). Since the disease prevalence exceeds 90% in female NOD-B7.2-/- mice and around 30% in males, female mice were exclusively utilized in these studies. Thirteen pre-symptomatic (*Pre*) NOD-B7.2-/- mice, aged 8-16 weeks and exhibiting no behavioral or electrophysiological signs of neuropathy, and thirteen symptomatic (*Sym*) SAPP mice, aged 24-36 weeks, were used to investigate endoneurial macrophages before and after disease onset, respectively.

### Neuromuscular severity score assessment

Clinical disability in NOD-B7.2-/- mice was assessed via a Neuromuscular Severity Score (NMSS). NMSS is based on the severity of muscle weakness and uses a 6-point scale: 0 – normal strength, 1 – mild tail weakness, 2 – mild/moderate fore or hind limb paresis, 3 – severe fore or hind limb paresis, 4 – mild/moderate tetraparesis, 5 – severe tetraparesis (29, 30).

### Electrophysiology

Sciatic nerve compound muscle action potential (CMAP) was measured as previously described (31, 32). Briefly, CMAP responses were recorded with needle electrodes inserted into the sole of the hind paw, while the sciatic nerves were stimulated proximally at the sciatic notch. A decrease in CMAP amplitude indicated axon degeneration, whereas proximal motor latency (ML) and CMAP duration were analyzed as electrophysiological indicators of demyelination (33).

### Sciatic nerve crush injury

Wild-type C57BL/6 mice (both male and female), aged 16-24 weeks, were used to generate the TPNI animal model through sciatic nerve crush injury. As previously described (31), bilateral sciatic nerve crush injury was induced 35 mm proximal to the toes with sterile blunt forceps for 60 seconds under isoflurane anesthesia. This was done bilaterally to ensure sufficient macrophage numbers, as pooling at least two sciatic nerves from each mouse was necessary to obtain adequate macrophage counts for flow cytometry analysis. To ensure the crush was successful, we visually checked for the disruption of the inner nerve fibers. Additionally, we measured the sciatic nerve CMAP responses three days after the procedure, and mice that still exhibited a CMAP response were excluded from further studies (one mouse was excluded due to an incomplete crush). Throughout the procedure, animals were closely monitored, and postoperative care was provided to minimize distress.

### Morphometry

Mice were euthanized via isoflurane overdosage and subsequently perfused with 1X phosphate-buffered saline (PBS). Tibial nerves from NOD-B7.2-/- mice in the SAPP model, as well as tibial nerve segments distal to the crush site in the TPNI animal model, were harvested. These nerve samples were then embedded in Epon, and 1µm cross sections were stained with toluidine blue. Subsequently, all myelinated axons within the cross-sections of the nerves were counted (31, 32). Demyelination was assessed by counting the number of completely demyelinated axons and thinly myelinated fibers in the entire cross-section of the nerve.

### Flow cytometry

Flow cytometry was performed on single cell suspensions from the sciatic nerves using a Beckman Coulter Cytoflex S flow cytometer (Beckman Coulter, USA). Mice were euthanized and perfused with PBS, and 1.5 cm sciatic nerve segments were harvested, starting 3 mm distal to the neuroforaminal exits to ensure consistent sampling across animals. For mice in the TPNI group, following the induction of crush injury as described above, we standardized the collection by excising the 1.5 cm nerve segment immediately distal to the lesion site in all animals. This uniform approach, with precise measurement from the lesion site, was employed to ensure comparability between nerve samples from different animals. Bilateral sciatic nerves from each mouse were pooled and mechanically processed by cutting the nerve segments into approximately 25 sections. These sections were then subjected to enzymatic digestion in a medium containing 10 mM HEPES, 5 mg/ml BSA, 1.6 mg/ml collagenase Type IV, and 100 μg/ml DNase in RPMI-1640. The digestion process involved two 20-minute incubations followed by a 10-minute incubation at 37°C with shaking at 100 rpm. After digestion, the resulting cell suspensions were filtered through 70 μm cell strainers (Corning, USA) and processed to generate single-cell suspensions, as described in previous studies (32, 34). To conduct staining of both extra- and intra-cellular antigens using specified antibodies, single-cell suspensions were first incubated with 1:800 Brefeldin A in RPMI-1640/10% fetal bovine serum (FBS) for two hours. Subsequently, the cells were stained with 1:1000 Zombie Aqua live/dead dye, followed by an incubation of 1:50 Fc blocking antibody. After washing, fluorochrome-conjugated extracellular antibody master mixture or fluorescence-minus-one (FMO) cocktails (diluted 1:50) were incubated with the cells for 20 minutes at room temperature (RT) in the dark. For intracellular staining, following fixation with IC Fixation Buffer (Invitrogen, USA) and permeabilization with Permeabilization Buffer (Invitrogen, USA), intracellular antibody master mixture or FMO cocktails (all diluted 1:25 in Permeabilization Buffer) were incubated with the cells for 30 min at RT in the dark. Finally, the cell suspensions were resuspended in 120 μl of cell staining buffer (CSB; Biolegend, USA) for flow cytometry analysis with an assimilated event count of 400,000 events per sample.

The endoneurial immune cell populations were analyzed as described (32). Sciatic nerve CD45^+^ leukocytes were initially divided into CD11b^+^ myeloid cells and CD11b^-^ lymphocytes. Within the myeloid population, subgroups included F4/80^-^/CD11c^+^ classical dendritic cells (cDCs) and F4/80^+^ macrophages. Macrophages were further categorized based on origin, polarization state, and cytokine profile. Origin-based categories comprised F4/80^int^ blood-derived macrophages (BDM) and F4/80^hi^ tissue-resident macrophages (TRM). Polarization states included CD80^+^/CD163^-^ M1 macrophages, CD80^+^/CD163^+^ intermediate-state macrophages, and CD80^-^/CD163^+^ M2 macrophages. Cytokine profiles differentiated IL-10^+^/TNF-α^-^ macrophages, IL-10^+^/TNF-α^+^ macrophages, and IL-10^-^/TNF-α^+^ macrophages. Among lymphocytes, CD4^+^ T-helper (Th) cells were identified and further subclassified into IL-10^+/-^/interferon-γ (IFN-γ)^+^ Th1 cells and IL-10^+^/IFN-γ^-^ Th2 cells. To determine the phagocytosis capacity of macrophages or cytokine production capacity of immune cells, we measured the median fluorescence intensities (MFI) of myelin protein 0 (MP0)-positive macrophages and cytokine-positive immune cells (32, 35). The gating strategy for identifying immune cell populations, myelin phagocytosis and cytokine production is outlined in **Supplementary Figure S1**. Flow cytometry data were analyzed using BD FlowJo 8.2.1 software.

### Statistical analysis

Numerical results are displayed as mean ± standard deviation (SD). Depending on Gaussian distribution, differences between two groups were investigated via unpaired t-test or Mann-Whitney U test. Multiple group analyses were either performed via analysis of variance (ANOVA) or Kruskal-Wallis test with corrections for multiple comparisons. *P*-values <0.05 were considered statistically significant. Flow cytometry cell population outliers derived from aberrant live or parental cell counts, exceeding or falling below the mean target cell counts by more than two SD, were excluded from analysis.

## Results

### Characterization of endoneurial immune responses and neuropathic phenotypes in mouse models of autoimmune polyneuropathy and traumatic nerve injury

**Figure 1:**
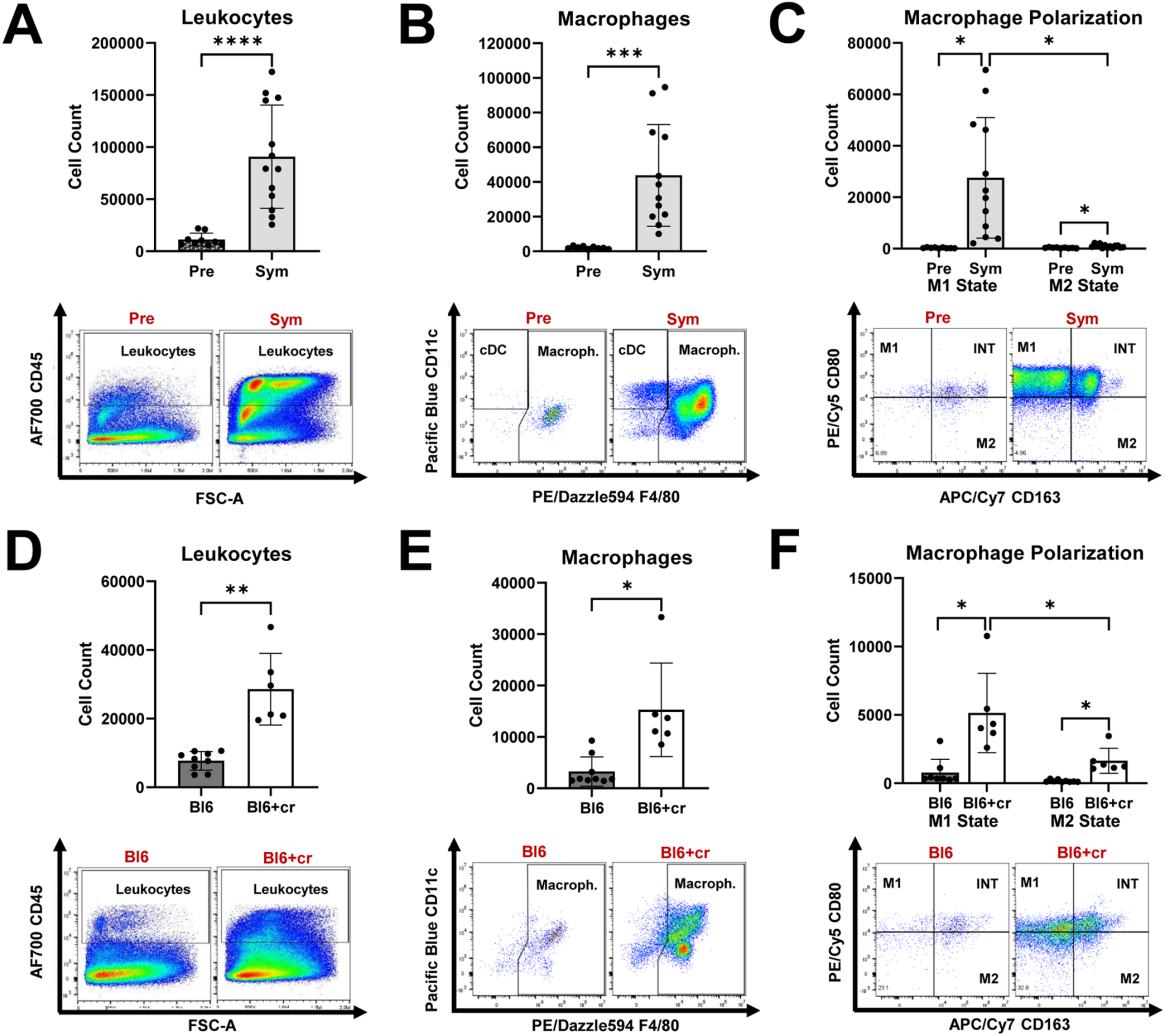
Endoneurial immune cell responses in mice with autoimmune polyneuropathy and traumatic peripheral nerve injury. **(A)** Symptomatic (*Sym*) NOD-B7.2-/- mice showed significantly increased endoneurial leukocyte counts than those of pre-symptomatic (*Pre*) mice. **(B)** Macrophages are significantly increased in *Sym* SAPP mice compared to *Pre* mice. **(C)** Both M1 and M2 macrophage counts are increased in *Sym* SAPP mice compared to *Pre* mice, with the majority being pro-inflammatory M1 macrophages. **(D)** Eight days after TPNI, *Bl6+cr* mice showed significantly increased endoneurial leukocyte counts than their naïve healthy counterparts (*Bl6*). **(E)** Significantly more macrophages were found in mice with TPNI. **(F)** Both M1 and M2 macrophages were increased eight days after TPNI, but in contrast to SAPP, the M1 polarization state was only about 2.5-times more abundant than the M2 state, indicating a more balanced macrophage polarization profile compared to in SAPP. SAPP, spontaneous autoimmune peripheral polyneuropathy, TPNI, traumatic peripheral nerve injury; Bl6, C57BL/6 mice without sciatic nerve crush; Bl6+cr, C57BL/6 mice with sciatic nerve crush; n =9-13 mice per group; *p<0.05, **p<0.01, ***p<0.001, ****p<0.0001.

First, by using flow cytometry, we assessed endoneurial immune cell activity in two models of nerve injury: autoimmune polyneuropathy (SAPP model) and traumatic peripheral nerve injury (sciatic nerve crush model). In *Sym* SAPP mice (*n=13*), we observed a significant increase in endoneurial leukocyte counts (**Figure 1A**), particularly macrophages, compared to *Pre* NOD-B7.2-/- mice (*n=13*) (**Figure 1B**; *Sym*: 43826 ± 29335 macrophages vs. *Pre*: 2098 ± 796 macrophages, *p=*0.0005). After disease onset in SAPP mice, both M1 and M2 macrophage populations increased remarkably. Notably, the pro-inflammatory M1 macrophages were predominant, being approximately 25-times more abundant than the M2 macrophages in *Sym* mice (**Figure 1C**). We further examined endoneurial immune cell responses following TPNI in wild-type C57BL/6 mice. Compared to healthy controls without TPNI (*Bl6, n=9*), C57BL/6 mice with sciatic nerve crush (*Bl6+cr, n=6*) showed a significant rise in both endoneurial leukocytes and macrophages at eight days post-injury (**Figures 1D, E**). We then analyzed the endoneurial macrophage polarization profile in the TPNI model and found that traumatic nerve injury induced both M1 and M2 polarization. Following TPNI, the macrophages were primarily M1 macrophages (**Figure 1F**). Interestingly, endoneurial M1 polarization was less pronounced in mice with TPNI compared to *Sym* SAPP mice, with M1 macrophages being only about 2.5 times more abundant than M2 macrophages in the nerves of mice with TPNI. Under baseline conditions (*Pre* SAPP mice and *Bl6* mice), most endoneurial macrophages exhibited an intermediate-state phenotype expressing CD80^+^CD163^+^ (refer to the dot plots in **Figures 1C, F**). Conversely, under neuropathic conditions (*Sym* SAPP mice and *Bl6+cr* mice), the M1 phenotype became the dominant macrophage type in both models, although the number of intermediate-state macrophages also increased (refer to the dot plots in **Figures 1C, F**).

In line with previous studies (29, 30), *Sym* SAPP mice mostly exhibited a moderate to severe flaccid tetraparesis as indicated by the NMSS score (mean NMSS: 4 ± 1, **Supplementary Figure S2A**). *Sym* SAPP mice manifested electrophysiologically and histologically as primarily demyelinating polyneuropathy with secondary axonal degeneration. Specifically, they showed reduced sciatic nerve CMAP amplitudes, increased CMAP durations, and prolonged proximal motor latencies compared to *Pre* counterparts (**Supplementary Figure S2A**). In the TPNI model, control *Bl6* mice without sciatic nerve crush demonstrated normal sciatic nerve CMAP amplitudes, whereas those in the crush group (*Bl6+cr*) showed sciatic nerve inexcitability at three- and seven-days post crush injury, indicating a complete nerve crush (**Supplementary Figure S2B**). Morphometric analysis of tibial nerve sections revealed that demyelination was significantly more severe in *Sym* SAPP mice than in *Pre* animals (**Supplementary Figure S2C**). Furthermore, a significant loss of myelinated fibers was observed in the tibial nerves of both *Sym* SAPP and TPNI mice eight days post-injury (*Bl6+cr*), compared to their respective controls (*Pre* or *Bl6*; **Supplementary Figures S2D, E**).

### Chronic pro-inflammatory macrophage responses in murine autoimmune polyneuropathy are primarily linked to tissue-resident macrophages within the nerve

**Figure 2:**
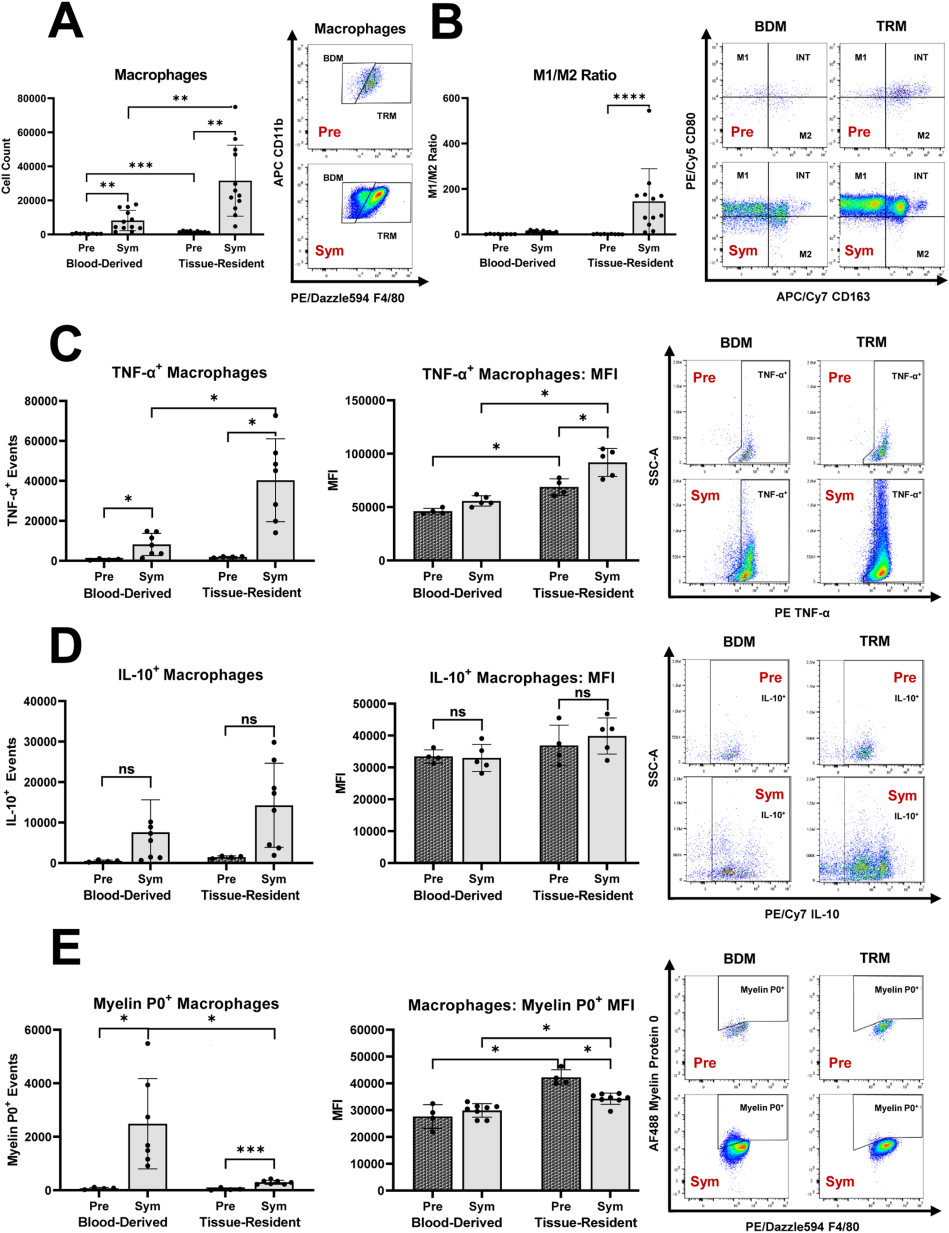
The distinct roles of tissue resident macrophages (TRM) and blood derived macrophages (BDM) within the endoneurium of mice with autoimmune polyneuropathy. **(A)** In sciatic nerves of pre-symptomatic NOD-B7.2-/- mice (*Pre*), the majority of endoneurial macrophages were of tissue-resident origin. Endoneurial macrophage increase in symptomatic SAPP (*Sym*) was mainly derived from the expansion of TRM. **(B)** S*ym* SAPP mice exhibited a significant pro-inflammatory shift in the M1/M2 macrophage polarization ratio among endoneurial TRM. **(C)** *Sym* SAPP mice exhibit a significantly increased TNF-α production by endoneurial macrophages compared to *Pre* mice. This increase is largely attributable to TRM. **(D)** There was no significant difference in the production of IL-10 by endoneurial BDM and TRM between *Sym* SAPP mice and *Pre* mice. **(E)** SAPP was associated with increased myelin protein 0 (MP0) phagocytosis, which was mainly carried out by newly recruited BDM. In contrast, TRM showed a higher per-cell phagocytic ability compared to BDM. SAPP, spontaneous autoimmune peripheral polyneuropathy; n=12 mice per group for cell surface marker assessments, n=5-8 mice per group for intracellular cytokine & MP0 assays; *p<0.05, **p<0.01, ***p<0.001, ****p<0.0001.

We demonstrated that *Sym* SAPP mice showed a marked increase in endoneurial macrophage counts compared to *Pre* mice (as shown in **Figure 1**). The endoneurial macrophage increase in SAPP was mainly derived from an expansion of TRM (CD11b^+^F4/80^hi^), which was significantly more pronounced than the increase in BDM (**Figure 2A**). Furthermore, an increase in pro-inflammatory M1 macrophages within the endoneurium was observed in *Sym* SAPP mice (*Sym*: 27552 ± 23425 vs. *Pre*: 313 ± 170 M1 macrophages, *p=*0.002; as shown in **Figure 1C**), with the majority of these M1 macrophages being of tissue-resident origin (refer to the dot plots in **Figure 2B**). We found a significant pro-inflammatory shift in the M1/M2 macrophage polarization ratio specifically and uniquely among the TRM, implying an association of TRM with the pro-inflammatory macrophage response in SAPP (**Figure 2B**). This finding was further supported by the assessment of cytokine profiles of BDM and TRM. *Sym* SAPP mice displayed a significantly elevated endoneurial TNF-α production compared to *Pre* mice, as indicated by both increased TNF-α^+^ event counts and higher per-cell TNF-α production, which was primarily attributed to TRM in the endoneurium (**Figure 2C**). However, there was no statistically significant difference in the production of anti-inflammatory IL-10 by endoneurial BDM and TRM between *Sym* SAPP mice and *Pre* mice (**Figure 2D**). This suggests a specific pro-inflammatory shift in the cytokine profile of expanding endoneurial TRM in *Sym* SAPP mice, characterized by higher TNF-α but relatively stable IL-10 production by TRM compared to the pre-symptomatic state.

Next, we examined the phagocytic activity of endoneurial macrophages in the SAPP model. In *Sym* SAPP mice, macrophage myelin protein 0 (MP0) phagocytosis was significantly enhanced compared to *Pre* mice, and the majority of MP0^+^ macrophage events in *Sym* SAPP mice were attributed to the BDM population (**Figure 2E**). Interestingly, we found that the per-cell MP0 phagocytic ability was highest for TRM in pre-symptomatic nerves (**Figure 2E**), likely due to the prolonged lifespan of these patrolling low-abundance antigen-presenting cells before undergoing apoptosis in a steady-state [3]. Although the per-cell phagocytic ability of TRM decreased after SAPP onset, TRM still retained their characteristic of increased per-cell phagocytic ability compared to newly recruited BDM (**Figure 2E**), as suggested in (25).

It can thus be postulated that in *Sym* SAPP mice, TRM serve as the primary macrophage subset responsible for creating a sustained pro-inflammatory endoneurial milieu, contributing to the chronic autoinflammation. Meanwhile, BDM, which are less unilaterally polarized, primarily engage in myelin phagocytosis, leading to demyelination of peripheral nerves.

### The acute endoneurial inflammation after TPNI is mainly orchestrated by blood-derived macrophages

**Figure 3:**
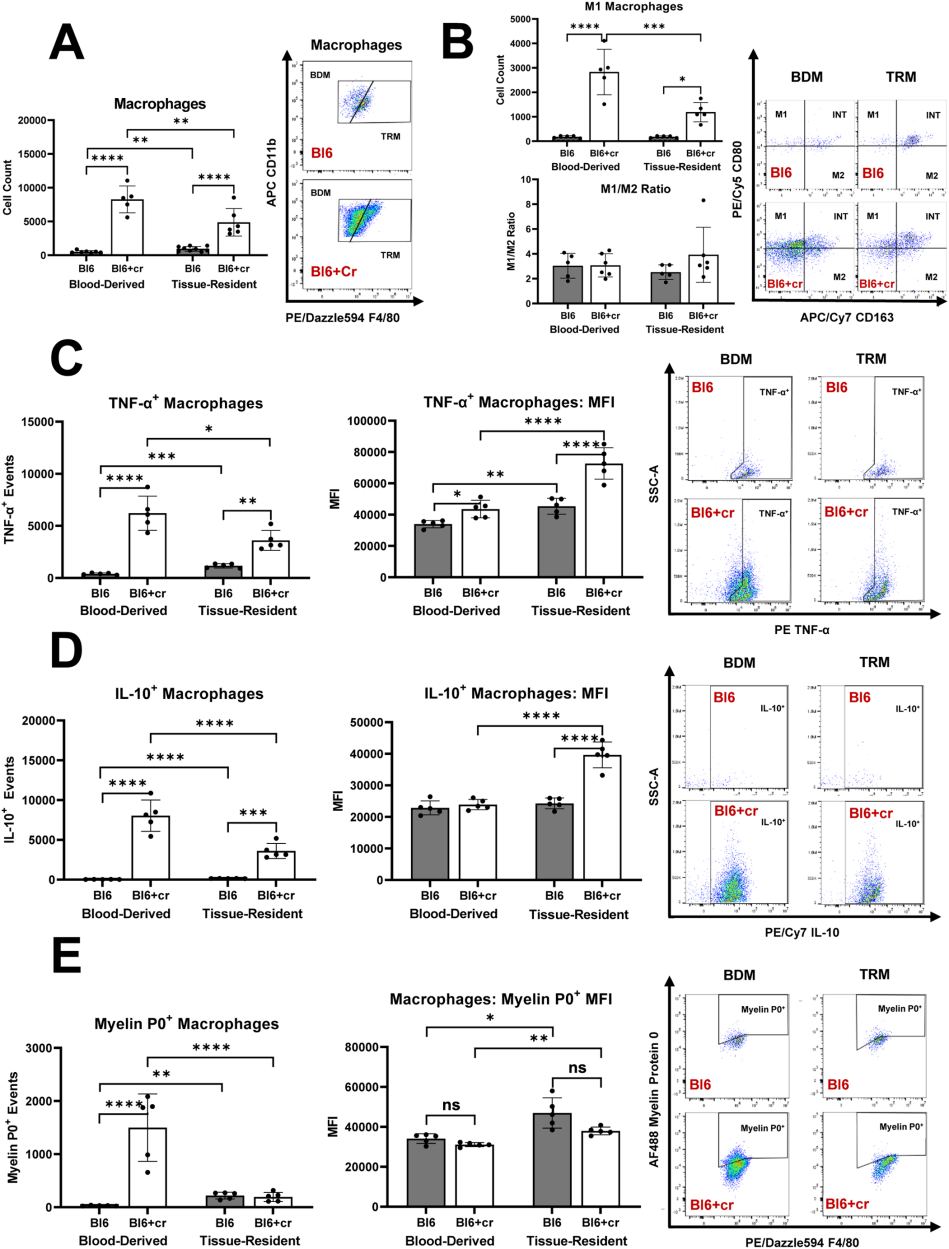
The distinct roles of tissue resident macrophages (TRM) and blood derived macrophages (BDM) within the endoneurium of mice with traumatic peripheral nerve injury (TPNI). **(A)** Eight days after bilateral sciatic nerve crush injury, newly recruited BDM became the major endoneurial macrophage population in C57BL/6 mice with sciatic nerve crush (*Bl6+cr*). Prior to injury, TRM constituted the majority of endoneurial macrophages. **(B)** After TPNI, both BDM and TRM showed a significant increase in M1 macrophage counts, with the majority of M1 macrophages being of blood-derived origin. There were no differences observed in the M1/M2 polarization ratio between controls (*Bl6*) and mice with TPNI (*Bl6+cr*). **(C)** Following TPNI, TNF-α^+^ events were significantly more abundant among both BDM and TRM. While BDM accounted for the majority of TNF-α^+^ and IL-10^+^ events, per-cell TNF-α production was higher in TRM compared to BDM. **(D)** After TPNI, IL-10^+^ events were significantly more abundant in BDM than TRM. However, per-cell production of IL-10 was higher in TRM than BDM. **(E)** Macrophage myelin protein 0 (P0) phagocytosis increased after TPNI, mainly carried out by BDM. However, endoneurial TRM exhibited higher per-cell phagocytic ability. n=5-9 mice per group, both male and female mice were included; *p<0.05, **p<0.01, ***p<0.001, ****p<0.0001.

In line with previous studies (1, 2), sciatic nerve crush injury led to significant increase in endoneurial macrophage counts eight days post-injury, with the majority of these macrophages being blood-derived (**Figure 3A**). There was a notable rise in endoneurial M1 macrophages in injured nerves, with most M1 macrophages also originating from the blood (**Figure 3B**). Notably, the balance between pro-inflammatory M1 and anti-inflammatory M2 macrophages differed significantly between TPNI mice and SAPP mice. Specifically, the predominant endoneurial macrophage subtype in TPNI mice (BDM) had a much lower M1/M2 ratios (mean ratio of 2-4) than the main endoneurial macrophage subset in *Sym* SAPP mice (TRM, mean ratio of 145). (*p*<0.0001; see **Figures 2B** vs. **3B**). This indicates a less pronounced pro-inflammatory macrophage polarization state following TPNI compared to SAPP. Next, we examined cytokine production by endoneurial macrophage subsets following nerve injury. We found that sciatic nerve crush resulted in a significant increase in TNF-α^+^ and IL-10^+^ production by endoneurial macrophages. TRM exhibited higher per-cell production of TNF-α and IL-10 cytokines than BDM after TPNI, although the majority of TNF-α^+^ and IL-10^+^ events were attributed to BDM (**Figures 3C, D**). This suggests that BDM played a more critical role than TRM in overall cytokine production during acute endoneurial inflammation following TPNI. After TPNI, endoneurial macrophages showed a more balanced cytokine profile, characterized by more equalized TNF-α and IL-10 production and lower overall TNF-α output compared to endoneurial macrophages in SAPP (compare **Figures 2C, D** with **Figures 3C, D**). Finally, similar to SAPP, phagocytosis of MP0 was mainly carried out by BDM, although TRM displayed a higher per-cell phagocytic ability (**Figure 3E**). There was no difference in per-cell phagocytic ability between healthy *Bl6* mice and the TPNI cohort (*Bl6+cr*) (**Figure 3E**).

### Effects of B7.2 knockout on endoneurial macrophages

First, we evaluated the potential impact of the transgenic B7.2 knockout on steady-state endoneurial macrophages in *Pre* NOD-B7.2-/- mice. Macrophage characteristics such as origins and polarization states were compared between *Pre* NOD-B7.2-/- (*n=*9; 16 weeks old) and C57BL/6 mice (*n=*9; 10-16 weeks old). No significant differences in endoneurial macrophage origins and polarization states were found between the two groups (**Supplementary Figure S3**).

**Figure 4:**
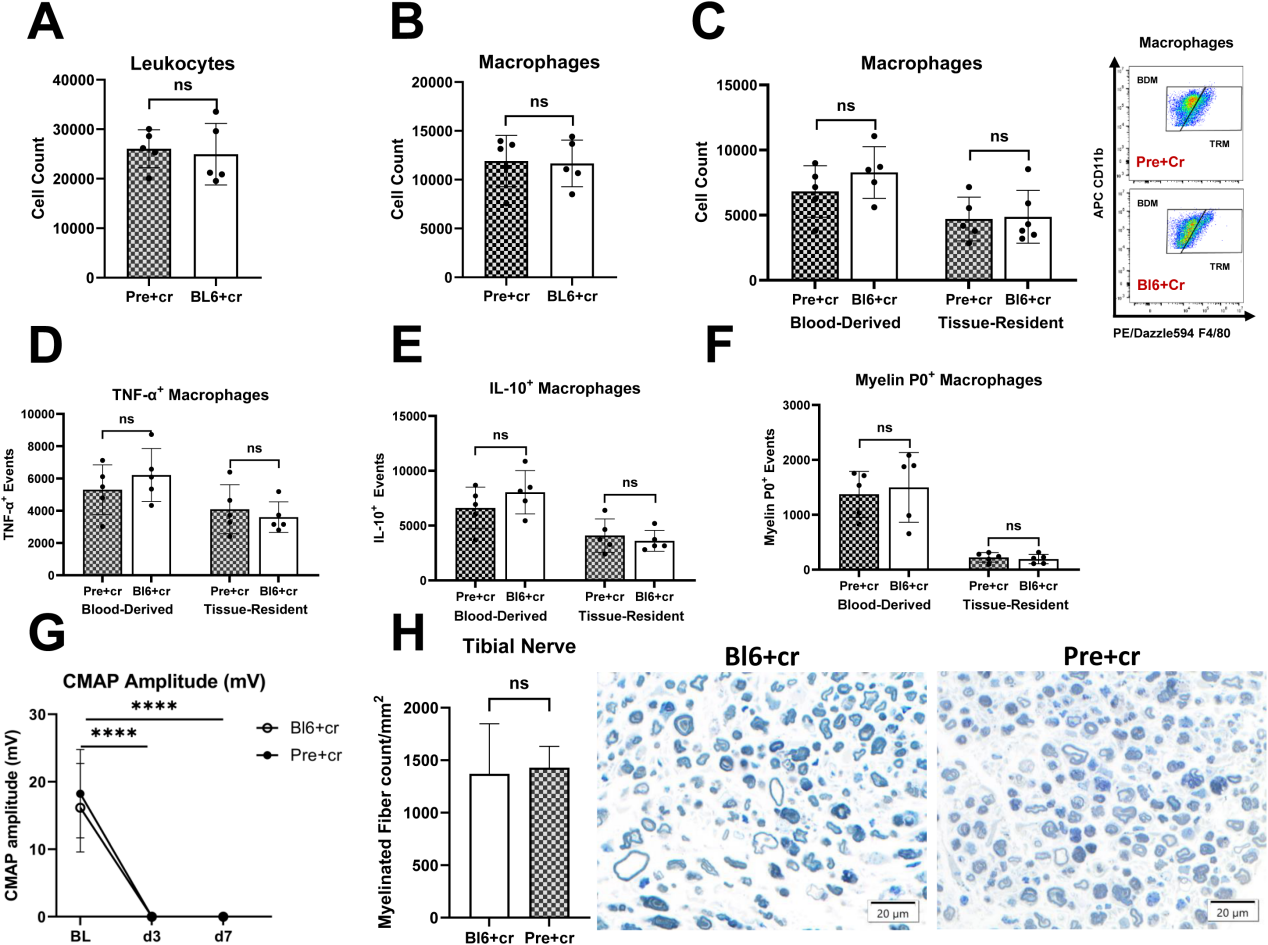
The endoneurial macrophage immune response after TPNI does not differ between pre-symptomatic NOD-B7.2-/- and C57BL/6 mice. **(A-F)** Eight days after sciatic nerve crush injury, the counts of endoneurial leukocytes **(A)**, macrophages **(B)**, BDM and TRM **(C)**, TNF-α^+^ macrophages **(D),** IL-10^+^ macrophages **(E)**, and myelin protein 0^+^ macrophages **(F)** were similar between pre-symptomatic NOD-B7.2-/- (Pre+cr) and C57BL/6 mice (BL6+cr). **(G, H)** Following TPNI, electrophysiological **(G)** and histological **(H)** outcomes were also comparable between these two genetically distinct mouse strains. n=5 mice per group. ns, not significant, ****p<0.0001.

We further examined whether the genetic alteration of B7.2 in NOD-B7.2-/- mice influences the origins of endoneurial macrophages following TPNI. Since the onset of SAPP could potentially affect the comparability between our NOD-B7.2-/- SAPP model and C57BL/6 TPNI model, we conducted bilateral sciatic nerve crush in *Pre* NOD-B7.2-/- (*n=*5; 16 weeks old) and C57BL/6 mice (*n=*5; 10-16 weeks old). We found no significant differences in endoneurial leukocyte and macrophage responses, macrophage origins, cytokine production by endoneurial macrophages, and endoneurial macrophage myelin phagocytosis between *Pre* NOD-B7.2-/- and C57BL/6 mice eight days after TPNI (**Figures 4A-F**). Additionally, electrophysiological and morphometric assessments showed no significant differences in post-crush axonal degeneration between the two groups. (**Figures 4G, H**). Collectively, our data suggest that the distinct roles of TRM and BDM observed in *Sym* NOD-B7.2-/- are characteristic of SAPP onset rather than an innate phenomenon independent of autoimmune polyneuropathy.

## Discussion

**Figure 5:**
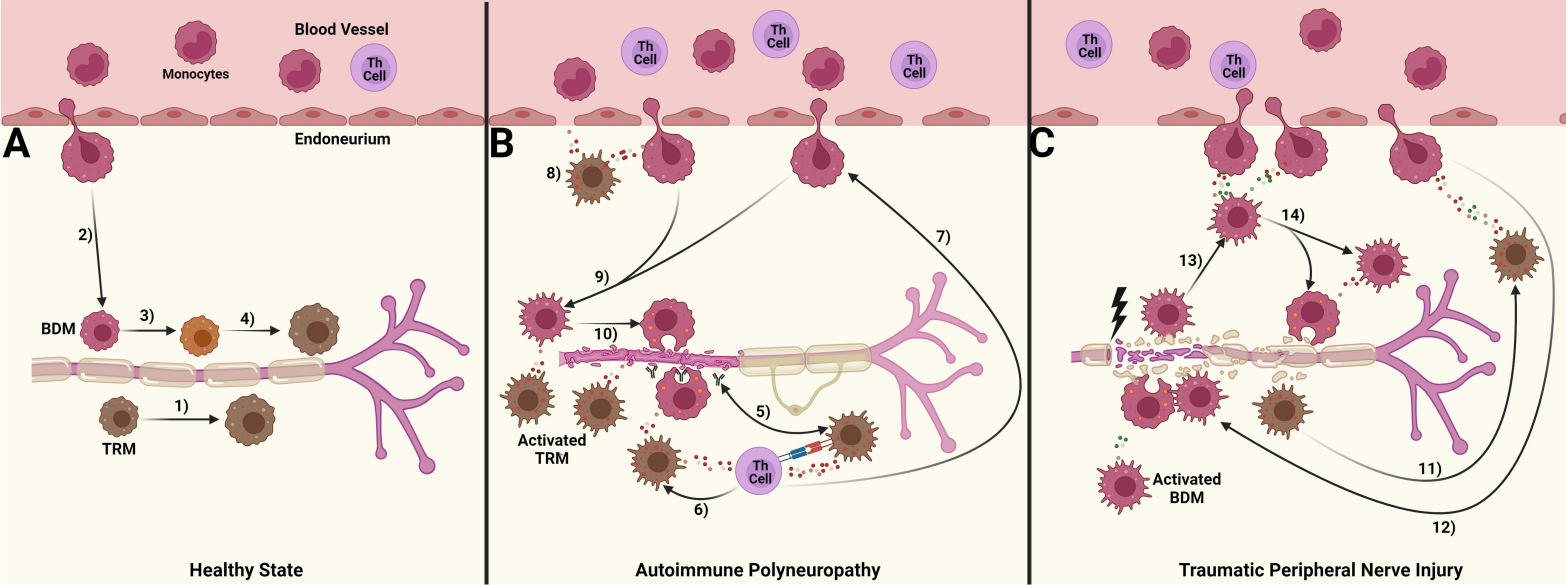
Suggested differential roles of blood-derived macrophages (BDM) and tissue resident macrophages (TRM) in autoimmune polyneuropathy and traumatic peripheral nerve injury. **(A)** In a healthy state, a small number of tissue-resident macrophages (TRM, *brown*) patrol the endoneurium. These cells are continuously replenished either by TRM-precursors (1) or through the limited migration of blood monocytes, which give rise to a population of blood-derived macrophages (BDM, *red*; 2). Over a period of time, the exact duration of which is still unknown, these BDMs acquire TRM markers (3, 4); **(B)** In chronic autoimmune polyneuropathy (spontaneous autoimmune peripheral polyneuropathy, SAPP), immune complexes formed by autoantibodies against myelin protein 0 (MP0) and MP0 are likely preferentially phagocytosed by TRM. This aberrant processing of MP0 may lead to its improper presentation to Th cells (5), which could *I)* induce and sustain pro-inflammatory cytokine signaling, further activating TRM and causing them to polarize towards a pro-inflammatory M1 state, thus promoting a persistent pro-inflammatory endoneurial milieu (6), and *II)* enhance the recruitment of BDM ( (7); possibly via CCL2 release), a process supported by TRM cytokine signaling (8). This influx of BDM drives the majority of myelin phagocytosis (9, 10), ultimately leading to peripheral nerve demyelination and secondary axonal degeneration; **(C)** Traumatic peripheral nerve injury (TPNI) after sciatic nerve crush is characterized by a more significant disruption of blood-nerve-barrier compared to SAPP, along with a dominant influx of BDM into the endoneurium. This influx is triggered by the transient TRM activation within 1-3 days after TPNI (11) and associated cytokine signaling. The cytokine response is more balanced between pro-and anti-inflammatory signals, aiming for a well-regulated immune response mediated by BDM (12–14). The pro-inflammatory M1 response of BDM peaks around 7-10 days after TPNI to clear endoneurial debris, before shifting towards a pro-regenerative M2 response to facilitate nerve recovery. This figure was designed with BioRender.com.

Our objective was to compare how macrophage responses in autoimmune polyneuropathy (AP) and traumatic peripheral nerve injury (TPNI) differ, to understand the different roles of endoneurial macrophages with various origins. Our study indicates that macrophage origin significantly impacts their function in these diseases, and these differences are evident between AP and TPNI (summarized in **Figure 5**). We here demonstrate that the endoneurial macrophage response in SAPP is mainly attributed to endoneurial TRM, whereas newly recruited BDMs exhibit less inflammatory characteristics compared to their TRM counterparts and mainly play a supporting role by executing phagocytosis. This finding extends the knowledge recently gained by a study using a conditional murine AP model [L31 mice (25)]. Oladiran and colleagues reported that the genetic knockout of CX3CR1, which is highly expressed by TRM and critical for their survival and proliferation, protected L31/CX3CR1-/- mice from immune-mediated polyneuropathy (25). However, the polyneuropathy was not prevented by knocking out CCR2 in this model. They postulated that since CCR2 is required for the transendothelial migration of BDM, the BDM recruitment into peripheral nerves may not be essential in AP. This is supported by their observation that in CCR2-/- mice, the expansion of CX3CR1^+^ endoneurial macrophages maintained the pro-inflammatory milieu, promoted myelin phagocytosis, and thus induced nerve damage.

Our findings indicate that in symptomatic SAPP mice, endoneurial TRM, but not BDM, exhibited a significant pro-inflammatory shift towards an M1 polarization state. This was accompanied by markedly increased TNF-α levels and stable IL-10 production, reinforcing the idea that TRM are the primary drivers of inflammation in AP. Our study used a SAPP model with spontaneous disease onset, in contrast to the L31 model where disease onset was initiated by contralateral sciatic nerve crush injury (25). This difference is important because nerve injury can trigger a systemic inflammatory response that might influence macrophage behavior. This explains the discrepancy between Oladrian et al.’s finding of a 1:1 ratio of F4/80^low/int^ BDM to F4/80^hi^ TRM in nerves of symptomatic L31 mice and our findings in the SAPP model showing significantly higher TRM than BDM levels using a similar gating strategy. Our spontaneous NOD-B7.2-/- SAPP model more closely resembles the inflammatory endoneurial macrophage response observed in CIDP.

It is currently unclear how long F4/80 expression remains at an intermediate level on newly recruited endoneurial BDM before transitioning to a high level similar to TRM. However, evidence on the dynamic change of Ly6C expression on BDM following TPNI (1) suggests that the macrophages identified as BDM by our flow cytometry analysis were present in the endoneurium for no more than 7 days. Our data indicate that in severe SAPP, the ongoing recruitment of BDM is less significant compared to the acute immune response to TPNI, and the expansion of endoneurial TRM is more crucial for maintaining the chronic autoimmune response in SAPP. Additionally, we observed that endoneurial TRM in SAPP mice showed significantly higher M1/M2 ratios and a more pro-inflammatory cytokine profile compared to endoneurial TRM in mice with TPNI, which exhibited a more balanced macrophage polarization and cytokine profile. This supports the notion that TRM are actively involved in orchestrating autoinflammation in AP. Furthermore, comparisons of endoneurial macrophage responses between pre-symptomatic NOD-B7.2-/- mice with TPNI and C57BL/6 mice with TPNI revealed a very similar macrophage response in both TPNI cohorts. This suggests that the pronounced pro-inflammatory TRM increase in SAPP is not a characteristic derived from the transgenic NOD-B7.2-/- background, as it does not manifest in pre-symptomatic animals with or without TPNI. Additionally, this TRM increase is not part of the typical “physiological” acute macrophage response to TPNI, which aims at clearing endoneurial debris to facilitate nerve repair. Moreover, our finding that TPNI, which primarily increases endoneurial BDM, failed to trigger an early onset of SAPP within eight days following nerve crush in pre-symptomatic NOD-B7.2-/- mice highlights the critical role of TRM expansion in SAPP development.

In contrast to the pronounced pro-inflammatory shift of TRM in SAPP, acute endoneurial inflammation eight days after TPNI was predominantly mediated by BDM. We chose to harvest nerve tissue at day 8 post TPNI, since pro-inflammatory macrophage infiltration peaks at this timepoint (2, 9). Our findings from the TPNI study align with previous studies, indicating a timely and predominantly blood-derived M1 macrophage response during the early stage (first week) after TPNI, followed by a shift towards a pro-regenerative M2 polarization state (9, 24).

In both SAPP and TPNI, phagocytosis was mainly carried out by newly recruited BDM. However, TRM displayed a higher per-cell phagocytic capacity in both models, with TRM in uninjured nerves showing the highest per-cell phagocytic ability. These findings are in line with previous studies which indicated that i) macrophage survival decreases under inflammatory conditions due to exposure to reactive oxygen species (3), and ii) CX3CR1, which is more abundant on TRM than on BDM, enhances the individual phagocytic ability of macrophages (25). Following TPNI, per-cell cytokine production was higher in TRM compared to BDM; however, due to the massive influx of BDM, most cytokine-positive events were attributed to BDM. TRM act as first responders within the first 1-3 days post-TPNI, initiating the orchestration of the inflammatory response. Subsequently, as indicated by our findings, further coordination of the injury response is mainly carried out by recruited BDM stimulated by CCL2 release from damaged neurons, Schwann cells and macrophages (2, 3). It remains to be evaluated whether BDM cytokine signaling suppresses the initial TRM response thereafter to prevent excessive endoneurial inflammation, potentially through IL-10 release. The absence of the CCL2 receptor, CCR2 (a BDM marker), hampers nerve recovery after peripheral nerve injury (36, 37), further underscoring the importance of BDM in peripheral nerve repair. In contrast, Oladiran and colleagues demonstrated that in AP, CX3CR1^+^ TRM compensate for the absence of BDM in L31/CCR2-/- mice and maintain endoneurial inflammation, reinforcing the crucial role of TRM in AP. This insight should be considered in the development of novel therapeutic approaches for AP including GBS and CIDP, which target macrophages and their recruitment to peripheral nerves (26). Systemic therapies may more readily reach blood-borne BDM precursors (monocytes) compared to TRM, particularly with moderate blood-nerve barrier disruption, potentially influencing treatment efficacy.

A limitation of our study is the current inability to clearly distinguish between long-term TRM and those derived from blood monocyte progenitors. Additionally, the kinetics by which newly recruited BDM acquire TRM characteristics, such as enhanced surface expression of F4/80 and/or CX3CR1 remain largely unexplored. Therefore, our study cannot reliably distinguish between established long-term TRM and replenished intermediate-term TRM. Although the differentiation of BDM and TRM based on F4/80 expression is a widely accepted method in nerve tissue and other organs (25, 38), it is important to acknowledge the limitation of this approach. Recent findings indicating a loss of the Ly6C marker on most BDM 5-7 days after their initial influx in response to TPNI (1) suggest that macrophage classified as “BDM” in our study likely entered the endoneurium less than a week prior to analysis, whereas TRM were likely present for at least seven days. Further investigations are warranted to comprehensively address this unresolved issue.

Additionally, future studies should systematically investigate how aging influences tissue-specific macrophage responses in both AP and TPNI models. In B7.2-/- NOD mice, endoneurial macrophage counts progressively increase until SAPP-associated death (29), which occurs at a median age of 40 weeks. This limits the comprehensive study of aging-related macrophage changes in these animals. However, comparing endoneurial macrophage responses in age- and sex-matched NOD wild-type mice would provide greater insight into age- and genetic background-related changes in endoneurial macrophages. To our knowledge, no other studies have focused on the temporal increase of TRM in SAPP during disease progression, but our findings suggest that macrophage expansion in the endoneurium during the disease course is primarily derived from TRM subsets. In mice with TPNI, studies have shown that aging increases endoneurial macrophage counts under steady-state conditions (a phenomenon referred to as “inflammaging”), followed by prolonged hyperinflammation in response to sciatic nerve crush injury (39). Additionally, a recent study from our group found that younger age is associated with increased endoneurial BDM M2 subsets three weeks after TPNI compared to aged mice, which promote neuroregeneration (32).

Sex is a crucial biological variable that influences immune responses, including the behavior of macrophages (40). Macrophages, essential for both the inflammatory and repair phases of nerve damage, exhibit sex-dependent differences in their activity due to the effects of sex hormones such as oestradiol, progesterone, and androgens. These hormones not only regulate immune cell recruitment and activation but also shape how macrophages behave in both inflammatory conditions, like AP, and in TPNI (41). These differences in macrophage responses driven by sex hormones influence key functions such as cytokine production, cell migration, phagocytosis, and overall macrophage activation status. The impact of these sex-based variations can be profound, as they could determine the extent of nerve damage following injury, the efficiency of nerve repair mechanisms, and even responses to therapeutic interventions. Future research aimed at unraveling the molecular mechanisms underlying these sex-specific macrophage responses is critical. A deeper understanding of how sex hormones regulate distinct macrophage subsets (e.g., TRM vs. BDM) in the context of nerve injury could open new avenues for targeted therapies.

Prior studies have demonstrated that endoneurial monocyte/macrophage infiltration is a hallmark of AP such as GBS and CIDP, as well as in TPNI (16, 42–44). Inflammatory leukocyte infiltration, particularly CD11b+ CD45+ macrophages, has been closely linked to disease severity in mouse models of both inflammatory and traumatic neuropathies, underscoring their role in pathogenesis (30, 45–47). Leukocyte trafficking, including that of monocytes and macrophages, into peripheral nerves is mediated by interactions between endothelial cells and leukocytes through selectins, chemokines, and adhesion molecules (16, 48, 49). While these studies have provided valuable insights into macrophage infiltration, our research builds on this knowledge by identifying and characterizing distinct subsets of endoneurial macrophages, TRM and BDM, in chronic endoneurial autoimmunity compared to the transient response seen in TPNI. This adds an important dimension to the understanding of macrophage biology in these conditions. We provide functional insights into how TRM and BDM differ in their roles across AP and TPNI models. Our findings emphasize the critical involvement of endoneurial TRM in the pathogenesis of AP. Unlike TPNI, which is marked by a predominant BDM response, AP is characterized by a robust TRM response, with a pronounced M1 pro-inflammatory phenotype. These TRM in AP show heightened production of TNF-α and likely play a more significant role in antigen presentation compared to those in TPNI, revealing origin-dependent differences in macrophage behavior, an issue that previously was not extensively explored. Moreover, our finding that myelin phagocytosis is primarily driven by BDM in both conditions offers new insights into the functional roles of these macrophage subsets.

These findings lay the groundwork for developing targeted therapies aimed at modulating specific macrophage subsets, which could result in more effective treatments for both AP and TPNI. Further research is necessary to fully elucidate the roles of TRM and BDM in peripheral neuropathies.

## Data Availability

The raw data supporting the conclusions of this article will be made available by the authors, without undue reservation.

## Supplementary material

Supplementary Figure 1 Flow cytometry gating strategy. Sciatic nerve leukocytes were gated as shown and then differentiated into myeloid and lymphoid cells based on CD11b expression. In a subgroup, CD4^+^ T helper (Th) cells were then distinguished from other lymphoid cells (*scattered arrow*). Myeloid cells were divided into F4/80^+^ macrophages and F4/80^-^CD11c^+^ classical dendritic cells (cDCs). Macrophages were sub-classified into F4/80^int^CD11b^hi^ blood-derived macrophages (BDM) and F4/80^hi^ tissue-resident macrophages (TRM). The M1/M2 macrophage polarization states, cumulative macrophage myelin protein 0 (MP0) phagocytosis, and macrophage cytokine production were assessed: **I)** CD80^+^CD163^-^ M1, CD80^+^CD163^+^ intermediate-state (INT), and CD80^-^CD163^+^ M2 macrophages; **II)** myelin P0^+^ macrophages; **III)** pro-inflammatory TNF-α^+^ and anti-inflammatory IL-10^+^ (event count and median fluorescence intensity, MFI).

Supplementary Figure 2 Behavioral, electrophysiological and histological outcomes in mice with autoimmune polyneuropathy and traumatic peripheral nerve injury. **(A)**: Symptomatic SAPP mice (*Sym, n*=13) showed clinical and electrophysiological signs of symmetrical axonal and demyelinating polyneuropathy, reflected by higher NMSS scores, decreased CMAP amplitudes, and increased CMAP duration and motor latency compared to pre-symptomatic NOD-B7.2-/- mice (*Pre, n*=13). **(B):** C57BL/6 mice with sciatic nerve crush injury (*Bl6+cr*; *n=6*; *both male and female mice were used*) showed sciatic nerve inexcitability 3- and 7-days post crush. **(C-E)**: Compared to *Pre* mice, *Sym* mice showed significantly more demyelination, as indicated by a higher g-ratio (**C**) and a decreased number of myelinated fibers in the tibial nerve **(D, E)**. Likewise, C57BL/6 Mice with TPNI (*Bl6+cr*) exhibited a significantly fewer tibial myelinated fibers than control C57BL/6 (*Bl6*) mice **(D, E)**. Arrow: demyelinated fibers; Arrow head: Onion-bulb formation; SAPP, spontaneous autoimmune peripheral polyneuropathy. ****p<0.0001.

Supplementary Figure 3 The effects of B7.2 knockout on steady state endoneurial macrophages in pre-symptomatic NOD-B7.2-/- mice. Macrophages and their polarization states in pre-symptomatic NOD-B7.2-/- (*Pre*) vs. C57BL/6 (*Bl6*) mice. n=9 mice per group. ns, not significant.

## References

[1] Ydens E Amann L Asselbergh B Scott CL Martens L Sichien D. Profiling peripheral nerve macrophages reveals two macrophage subsets with distinct localization, transcriptome and response to injury. Nat Neurosci. (2020) 23:676–89. doi: 10.1038/s41593-020-0618-6 PMC7611025 32284604

[2] Mueller M Leonhard C Wacker K Ringelstein EB Okabe M Hickey WF. Macrophage response to peripheral nerve injury: the quantitative contribution of resident and hematogenous macrophages. Lab Invest. (2003) 83:175–85. doi: 10.1097/01.lab.0000056993.28149.bf 12594233

[3] Msheik Z El Massry M Rovini A Billet F Desmouliere A. The macrophage: A key player in the pathophysiology of peripheral neuropathies. J Neuroinflamm. (2022) 19:97. doi: 10.1186/s12974-022-02454-6 PMC9013246 35429971

[4] Kiefer R Kieseier BC Stoll G Hartung HP. The role of macrophages in immune-mediated damage to the peripheral nervous system. Prog Neurobiol. (2001) 64:109–27. doi: 10.1016/s0301-0082(00)00060-5 11240209

[5] Iwai H Ataka K Suzuki H Dhar A Kuramoto E Yamanaka A. Tissue-resident M2 macrophages directly contact primary sensory neurons in the sensory ganglia after nerve injury. J Neuroinflamm. (2021) 18:227. doi: 10.1186/s12974-021-02283-z PMC8513227 34645458

[6] Cattin AL Burden JJ Van Emmenis L Mackenzie FE Hoving JJ Garcia Calavia N. Macrophage-induced blood vessels guide schwann cell-mediated regeneration of peripheral nerves. Cell. (2015) 162:1127–39. doi: 10.1016/j.cell.2015.07.021 PMC4553238 26279190

[7] Golshadi M Claffey EF Grenier JK Miller A Willand M Edwards MG. Delay modulates the immune response to nerve repair. NPJ Regener Med. (2023) 8:12. doi: 10.1038/s41536-023-00285-4 PMC9970988 36849720

[8] Zigmond RE Echevarria FD. Macrophage biology in the peripheral nervous system after injury. Prog Neurobiol. (2019) 173:102–21. doi: 10.1016/j.pneurobio.2018.12.001 PMC6340791 30579784

[9] Liu P Peng J Han GH Ding X Wei S Gao G. Role of macrophages in peripheral nerve injury and repair. Neural Regener Res. (2019) 14:1335–42. doi: 10.4103/1673-5374.253510 PMC6524518 30964051

[10] Lehmann HC Burke D Kuwabara S. Chronic inflammatory demyelinating polyneuropathy: update on diagnosis, immunopathogenesis and treatment. J Neurol Neurosurg Psychiatry. (2019) 90:981–7. doi: 10.1136/jnnp-2019-320314 30992333

[11] Shahrizaila N Lehmann HC Kuwabara S. Guillain-barre syndrome. Lancet. (2021) 397:1214–28. doi: 10.1016/S0140-6736(21)00517-1 33647239

[12] Sheikh KA Zhang G. An update on pathobiologic roles of anti-glycan antibodies in guillain-barre syndrome. F1000 Biol Rep. (2010) 2. doi: 10.3410/B2-21 PMC2948347 20948812

[13] Sheikh KA. Guillain-barre syndrome. Continuum (Minneap Minn). (2020) 26:1184–204. doi: 10.1212/CON.0000000000000929 33002998

[14] Klehmet J Goehler J Ulm L Kohler S Meisel C Meisel A. Effective treatment with intravenous immunoglobulins reduces autoreactive T-cell response in patients with cidp. J Neurol Neurosurg Psychiatry. (2015) 86:686–91. doi: 10.1136/jnnp-2014-307708 25074566

[15] Svacina MKR Meissner A Schweitzer F Ladwig A Pitarokoili K Kofler DM. Immunomodulatory effects of intravenous and subcutaneous immunoglobulin in chronic inflammatory demyelinating polyneuropathy: an observational study. Eur J Neurol. (2024) 31:e16079. doi: 10.1111/ene.16079 37789648 PMC11235934

[16] Ubogu EE. Inflammatory neuropathies: pathology, molecular markers and targets for specific therapeutic intervention. Acta Neuropathol. (2015) 130:445–68. doi: 10.1007/s00401-015-1466-4 PMC4575885 26264608

[17] Sukenikova L Mallone A Schreiner B Ripellino P Nilsson J Stoffel M. Autoreactive T cells target peripheral nerves in guillain-barre syndrome. Nature. (2024) 626:160–8. doi: 10.1038/s41586-023-06916-6 PMC10830418 38233524

[18] Beppu M Sawai S Misawa S Sogawa K Mori M Ishige T. Serum cytokine and chemokine profiles in patients with chronic inflammatory demyelinating polyneuropathy. J Neuroimmunol. (2015) 279:7–10. doi: 10.1016/j.jneuroim.2014.12.017 25669993

[19] Andersson J Skansen-Saphir U Sparrelid E Andersson U. Intravenous immune globulin affects cytokine production in T lymphocytes and monocytes/macrophages. Clin Exp Immunol. (1996) 104 Suppl 1:10–20. 8625537

[20] Koike H Nishi R Ikeda S Kawagashira Y Iijima M Katsuno M. Ultrastructural mechanisms of macrophage-induced demyelination in cidp. Neurology. (2018) 91:1051–60. doi: 10.1212/WNL.0000000000006625 30429275

[21] Hartung HP Toyka KV. T-cell and macrophage activation in experimental autoimmune neuritis and guillain-barre syndrome. Ann Neurol. (1990) 27 Suppl:S57–63. doi: 10.1002/ana.410270716 2194429

[22] Shen D Chu F Lang Y Geng Y Zheng X Zhu J. Beneficial or harmful role of macrophages in guillain-barre syndrome and experimental autoimmune neuritis. Mediators Inflammation. (2018) 2018:4286364. doi: 10.1155/2018/4286364 PMC5944239 29853789

[23] Koike H Katsuno M. Macrophages and autoantibodies in demyelinating diseases. Cells. (2021) 10. doi: 10.3390/cells10040844 PMC8068327 33917929

[24] Mokarram N Merchant A Mukhatyar V Patel G Bellamkonda RV. Effect of modulating macrophage phenotype on peripheral nerve repair. Biomaterials. (2012) 33:8793–801. doi: 10.1016/j.biomaterials.2012.08.050 PMC3483037 22979988

[25] Oladiran O Shi XQ Fournier S Zhang J. Cx3cr1 but not ccr2 expression is required for the development of autoimmune peripheral neuropathy in mice. Front Immunol. (2021) 12:720733. doi: 10.3389/fimmu.2021.720733 34484228 PMC8415420

[26] Shen D Lang Y Chu F Wu X Wang Y Zheng X. Roles of macrophage migration inhibitory factor in guillain-barre syndrome and experimental autoimmune neuritis: beneficial or harmful? Expert Opin Ther Targets. (2018) 22:567–77. doi: 10.1080/14728222.2018.1484109 29856236

[27] Shen D Chu F Lang Y Zheng C Li C Liu K. Nuclear factor kappa B inhibitor suppresses experimental autoimmune neuritis in mice via declining macrophages polarization to M1 type. Clin Exp Immunol. (2021) 206:110–7. doi: 10.1111/cei.13637 PMC8446411 34118070

[28] Salomon B Rhee L Bour-Jordan H Hsin H Montag A Soliven B. Development of spontaneous autoimmune peripheral polyneuropathy in B7-2-deficient nod mice. J Exp Med. (2001) 194:677–84. doi: 10.1084/jem.194.5.677 PMC2195945 11535635

[29] Ubogu EE Yosef N Xia RH Sheikh KA. Behavioral, electrophysiological, and histopathological characterization of a severe murine chronic demyelinating polyneuritis model. J Peripher Nerv Syst. (2012) 17:53–61. doi: 10.1111/j.1529-8027.2012.00375.x 22462666 PMC5792059

[30] Zhang G Bogdanova N Gao T Sheikh KA. Elimination of activating fcgamma receptors in spontaneous autoimmune peripheral polyneuropathy model protects from neuropathic disease. PloS One. (2019) 14:e0220250. doi: 10.1371/journal.pone.0220250 31415574 PMC6695161

[31] Lehmann HC Lopez PH Zhang G Ngyuen T Zhang J Kieseier BC. Passive immunization with anti-ganglioside antibodies directly inhibits axon regeneration in an animal model. J Neurosci. (2007) 27:27–34. doi: 10.1523/JNEUROSCI.4017-06.2007 17202469 PMC6672271

[32] Svacina MKR Gao T Sprenger-Svacina A Lin J Ganesh BP Lee J. Rejuvenating fecal microbiota transplant enhances peripheral nerve repair in aged mice by modulating endoneurial inflammation. Exp Neurol. (2024) 376:114774. doi: 10.1016/j.expneurol.2024.114774 38599367

[33] Tankisi H Otto M Pugdahl K Johnsen B Fuglsang-Frederiksen A. Correlation between compound muscle action potential amplitude and duration in axonal and demyelinating polyneuropathy. Clin Neurophysiol. (2012) 123:2099–105. doi: 10.1016/j.clinph.2012.04.002 22560637

[34] Hidmark AS Nawroth PP Fleming T. Analysis of immune cells in single sciatic nerves and dorsal root ganglion from a single mouse using flow cytometry. J Vis Exp. (2017) (130). doi: 10.3791/56538 PMC5755528 29286468

[35] Ritzel RM Li Y Jiao Y Lei Z Doran SJ He J. Brain injury accelerates the onset of a reversible age-related microglial phenotype associated with inflammatory neurodegeneration. Sci Adv. (2023) 9:eadd1101. doi: 10.1126/sciadv.add1101 36888713 PMC9995070

[36] Pan D Acevedo-Cintron JA Sayanagi J Snyder-Warwick AK Mackinnon SE Wood MD. The ccl2/ccr2 axis is critical to recruiting macrophages into acellular nerve allograft bridging a nerve gap to promote angiogenesis and regeneration. Exp Neurol. (2020) 331:113363. doi: 10.1016/j.expneurol.2020.113363 32450192 PMC7484126

[37] Lu CY Santosa KB Jablonka-Shariff A Vannucci B Fuchs A Turnbull I. Macrophage-derived vascular endothelial growth factor-a is integral to neuromuscular junction reinnervation after nerve injury. J Neurosci. (2020) 40:9602–16. doi: 10.1523/JNEUROSCI.1736-20.2020 PMC7726545 33158964

[38] Santamaria-Barria JA Zeng S Greer JB Beckman MJ Seifert AM Cohen NA. Csf1r or mer inhibition delays liver regeneration via suppression of kupffer cells. PloS One. (2019) 14:e0216275. doi: 10.1371/journal.pone.0216275 31042769 PMC6493758

[39] Buttner R Schulz A Reuter M Akula AK Mindos T Carlstedt A. Inflammaging impairs peripheral nerve maintenance and regeneration. Aging Cell. (2018) 17:e12833. doi: 10.1111/acel.12833 30168637 PMC6260910

[40] Klein SL Flanagan KL. Sex differences in immune responses. Nat Rev Immunol. (2016) 16:626–38. doi: 10.1038/nri.2016.90 27546235

[41] Szabo-Pardi TA Syed UM Castillo ZW Burton MD. Use of integrated optical clearing and 2-photon imaging to investigate sex differences in neuroimmune interactions after peripheral nerve injury. Front Cell Dev Biol. (2021) 9:624201. doi: 10.3389/fcell.2021.624201 34178976 PMC8221108

[42] Liu L Yin Y Li F Malhotra C Cheng J. Flow cytometry analysis of inflammatory cells isolated from the sciatic nerve and drg after chronic constriction injury in mice. J Neurosci Methods. (2017) 284:47–56. doi: 10.1016/j.jneumeth.2017.04.012 28445708

[43] Ristoiu V. Contribution of macrophages to peripheral neuropathic pain pathogenesis. Life Sci. (2013) 93:870–81. doi: 10.1016/j.lfs.2013.10.005 24140886

[44] Dong C Palladino SP Helton ES Ubogu EE. The pathogenic relevance of alpha(M)-integrin in guillain-barre syndrome. Acta Neuropathol. (2016) 132:739–52. doi: 10.1007/s00401-016-1599-0 PMC5074851 27460017

[45] Xia RH Yosef N Ubogu EE. Clinical, electrophysiological and pathologic correlations in a severe murine experimental autoimmune neuritis model of guillain-barre syndrome. J Neuroimmunol. (2010) 219:54–63. doi: 10.1016/j.jneuroim.2009.11.019 20034679

[46] Dong C Ubogu EE. Pro-inflammatory cytokines and leukocyte integrins associated with chronic neuropathic pain in traumatic and inflammatory neuropathies: initial observations and hypotheses. Front Immunol. (2022) 13:935306. doi: 10.3389/fimmu.2022.935306 35983047 PMC9378781

[47] Dong C Greathouse KM Beacham RL Palladino SP Helton ES Ubogu EE. Fibronectin connecting segment-1 peptide inhibits pathogenic leukocyte trafficking and inflammatory demyelination in experimental models of chronic inflammatory demyelinating polyradiculoneuropathy. Exp Neurol. (2017) 292:35–45. doi: 10.1016/j.expneurol.2017.02.012 28215575 PMC5400680

[48] Muller WA. Mechanisms of leukocyte transendothelial migration. Annu Rev Pathol. (2011) 6:323–44. doi: 10.1146/annurev-pathol-011110-130224 PMC3628537 21073340

[49] Simon SI Green CE. Molecular mechanics and dynamics of leukocyte recruitment during inflammation. Annu Rev BioMed Eng. (2005) 7:151–85. doi: 10.1146/annurev.bioeng.7.060804.100423 16004569

