# Supplementary figures and images for: Differential regulation of tissue-resident and blood-derived macrophages in models of autoimmune and traumatic peripheral nerve injury
Source: Front Immunol. 2024 Nov 19;15:1487788. doi: 10.3389/fimmu.2024.1487788 (PMC11611839; doi:10.3389/fimmu.2024.1487788)

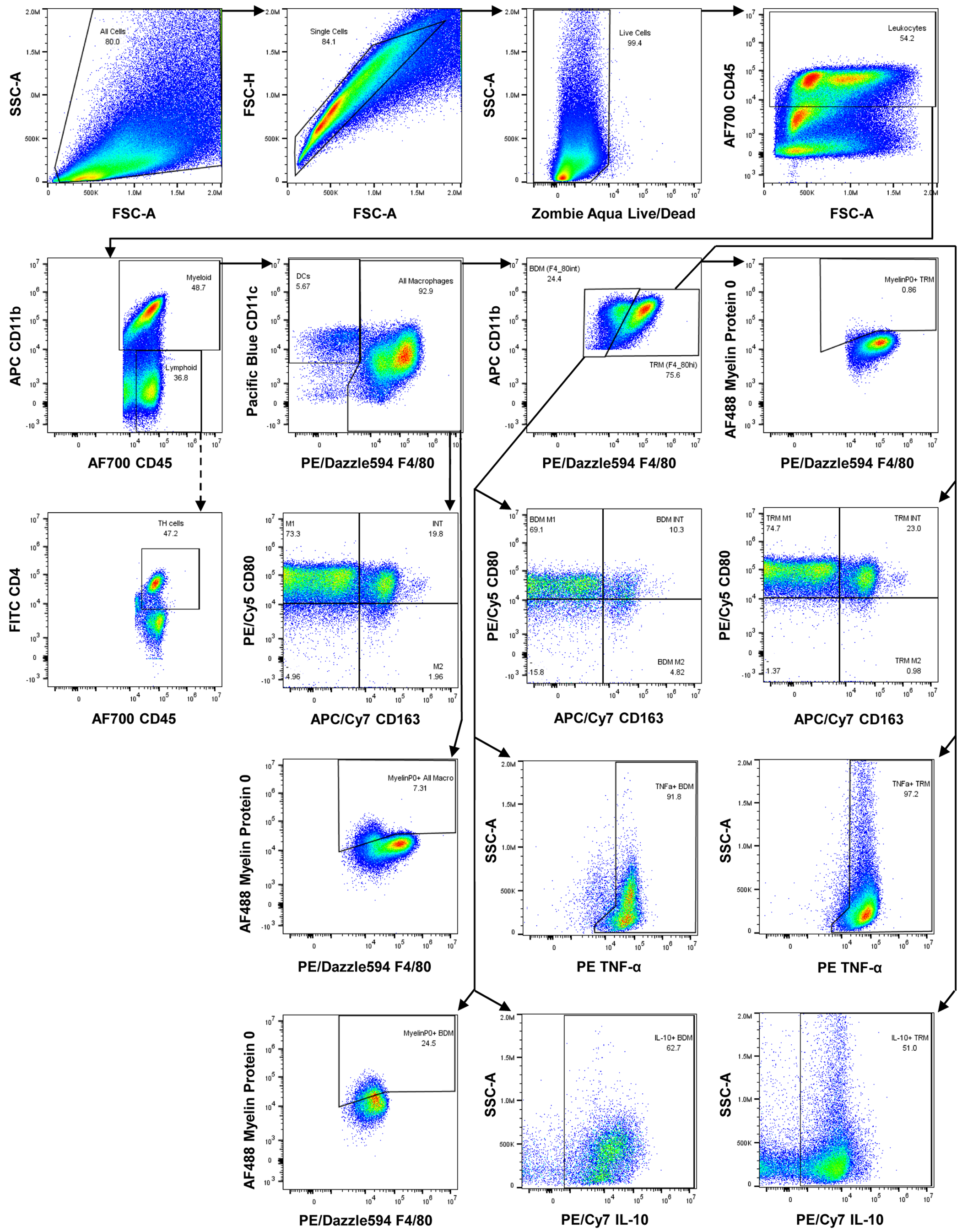

Supplement: Supplementary Figure 1 — Flow cytometry gating strategy. Sciatic nerve leukocytes were gated as shown and then differentiated into myeloid and lymphoid cells based on CD11b expression. In a subgroup, CD4+ T helper (Th) cells were then distinguished from other lymphoid cells (scattered arrow). Myeloid cells were divided into F4/80+ macrophages and F4/80-CD11c+ classical dendritic cells (cDCs). Macrophages were sub-classified into F4/80intCD11bhi blood-derived macrophages (BDM) and F4/80hi tissue-resident macrophages (TRM). The M1/M2 macrophage polarization states, cumulative macrophage myelin protein 0 (MP0) phagocytosis, and macrophage cytokine production were assessed: I) CD80+CD163- M1, CD80+CD163+ intermediate-state (INT), and CD80-CD163+ M2 macrophages; II) myelin P0+ macrophages; III) pro-inflammatory TNF-α+ and anti-inflammatory IL-10+ (event count and median fluorescence intensity, MFI). [file Image1.tif]

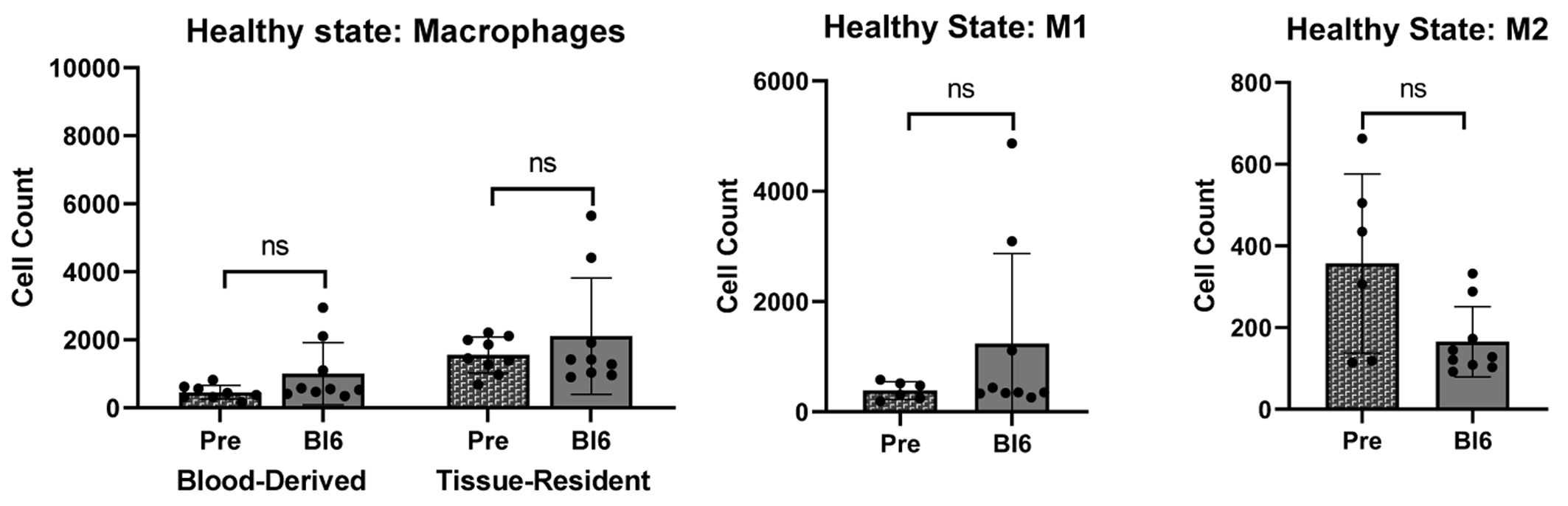

Supplement: Supplementary Figure 2 — Behavioral, electrophysiological and histological outcomes in mice with autoimmune polyneuropathy and traumatic peripheral nerve injury. (A): Symptomatic SAPP mice (Sym, n=13) showed clinical and electrophysiological signs of symmetrical axonal and demyelinating polyneuropathy, reflected by higher NMSS scores, decreased CMAP amplitudes, and increased CMAP duration and motor latency compared to pre-symptomatic NOD-B7.2-/- mice (Pre, n=13). (B): C57BL/6 mice with sciatic nerve crush injury (Bl6+cr; n=6; both male and female mice were used) showed sciatic nerve inexcitability 3- and 7-days post crush. (C-E): Compared to Pre mice, Sym mice showed significantly more demyelination, as indicated by a higher g-ratio (C) and a decreased number of myelinated fibers in the tibial nerve (D, E). Likewise, C57BL/6 Mice with TPNI (Bl6+cr) exhibited a significantly fewer tibial myelinated fibers than control C57BL/6 (Bl6) mice (D, E). Arrow: demyelinated fibers; Arrow head: Onion-bulb formation; SAPP, spontaneous autoimmune peripheral polyneuropathy. ****p<0.0001. [file Image2.tif]

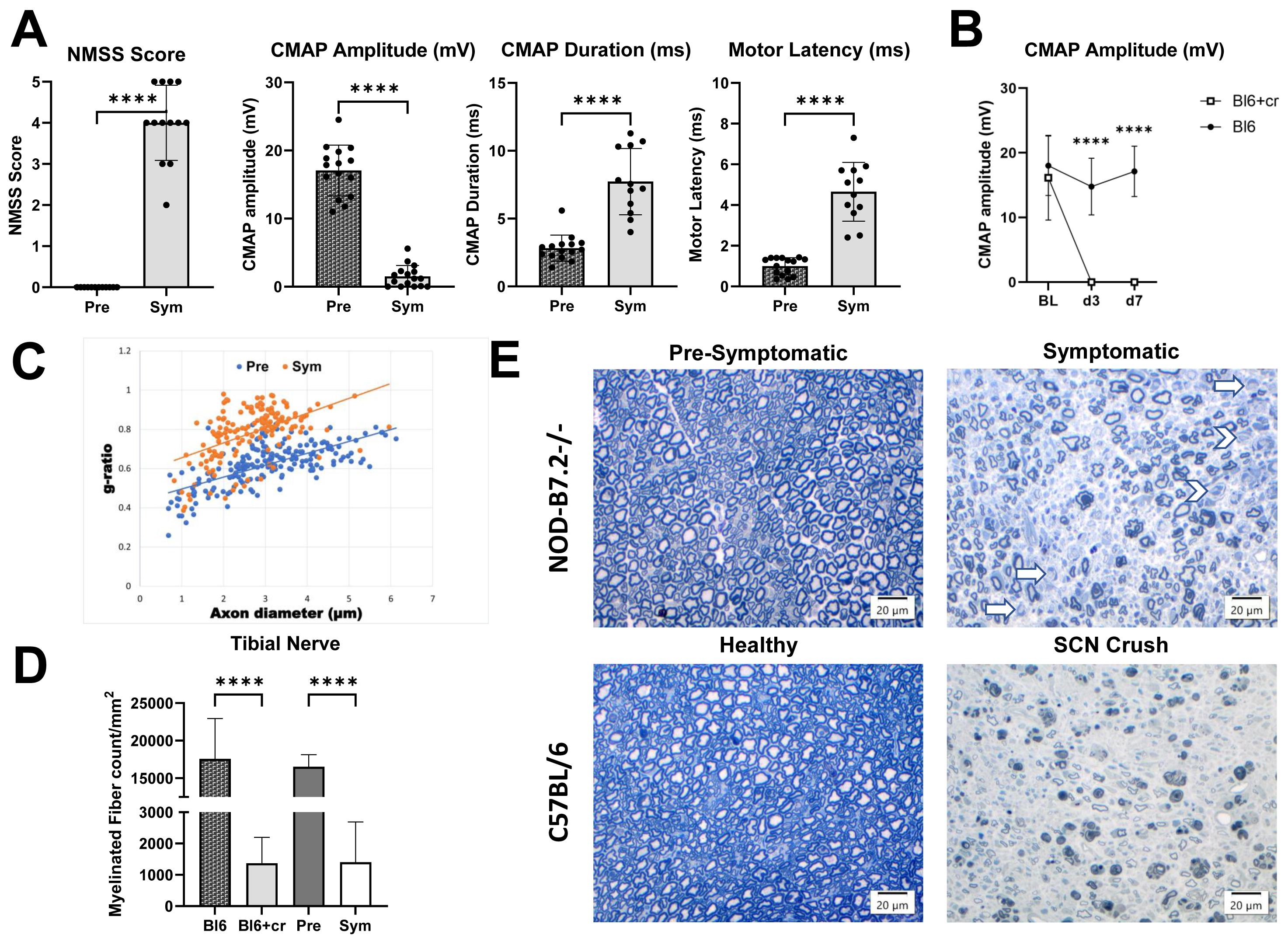

Supplement: Supplementary Figure 3 — The effects of B7.2 knockout on steady state endoneurial macrophages in pre-symptomatic NOD-B7.2-/- mice. Macrophages and their polarization states in pre-symptomatic NOD-B7.2-/- (Pre) vs. C57BL/6 (Bl6) mice. n=9 mice per group. ns, not significant. [file Image3.tif]
